# Acrylates Polymerization on Covalent Plasma-Assisted Functionalized Graphene: A Route to Synthesize Hybrid Functional Materials

**DOI:** 10.1021/acsami.3c07200

**Published:** 2023-09-22

**Authors:** Roberto Muñoz, Laia León-Boigues, Elena López-Elvira, Carmen Munuera, Luis Vázquez, Federico Mompeán, José Ángel Martín-Gago, Irene Palacio, Mar García-Hernández

**Affiliations:** † Instituto de Ciencia de Materiales de Madrid (ICMM), CSIC, Sor Juana Inés de la Cruz 3, Madrid E-28049, Spain; ‡ Universidad Complutense de Madrid, Madrid E-28040, Spain

**Keywords:** hybrid materials, functionalization, graphene, plasma, polymer, acrylates

## Abstract

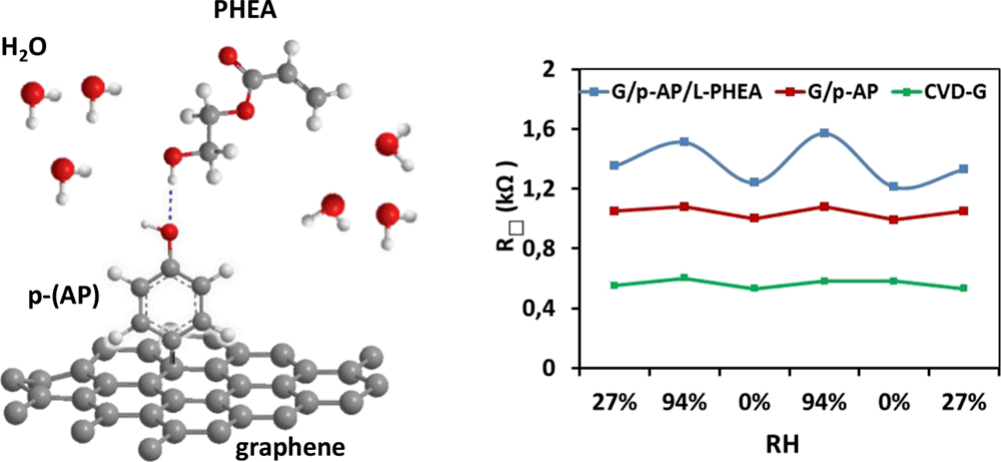

The modification of the surface properties of graphene with polymers provides a method for expanding its scope into new applications as a hybrid material. Unfortunately, the chemical inertness of graphene hinders the covalent functionalization required to build them up. Developing new strategies to enhance the graphene chemical activity for efficient and stable functionalization, while preserving its electronic properties, is a major challenge. We here devise a covalent functionalization method that is clean, reproducible, scalable, and technologically relevant for the synthesis of a large-scale, substrate-supported graphene–polymer hybrid material. In a first step, hydrogen-assisted plasma activation of *p*-aminophenol (*p*-AP) linker molecules produces their stable and covalent attachment to large-area graphene. Second, an in situ radical polymerization reaction of 2-hydroxyethyl acrylate (HEA) is carried out on the functionalized surface, leading to a graphene–polymer hybrid functional material. The functionalization with a hydrophilic and soft polymer modifies the hydrophobicity of graphene and might enhance its biocompatibility. We have characterized these hybrid materials by atomic force microscopy (AFM), X-Ray photoelectron spectroscopy (XPS) and Raman spectroscopy and studied their electrical response, confirming that the graphene/*p*-AP/PHEA architecture is anchored covalently by the sp^3^ hybridization and controlled polymerization reaction on graphene, retaining its suitable electronic properties. Among all the possibilities, we assess the proof of concept of this graphene-based hybrid platform as a humidity sensor. An enhanced sensitivity is obtained in comparison with pristine graphene and related materials. This functional nanoarchitecture and the two-step strategy open up future potential applications in sensors, biomaterials, or biotechnology fields.

## 1 Introduction

Graphene, an sp^2^ carbon lattice, exhibits unique properties such as extreme in-plane electron mobility and intrinsic chemical inertness.^1,2^ This appealing chemical stability, however, can be an issue when the intended application requires a robust and stable graphene combination with other molecules or materials, mainly by covalent bonding, as in hybrid–polymer platforms for sensing and signal transduction.^3,4^ Nevertheless, the significant influence of covalent functionalization in the transport properties of graphene is well established.^5^ Diverse graphene functionalization approaches have been devised for years, addressing robust attachment requirements while maintaining the high conductivity of the graphene layer.^3,6−8^ The most common covalent functionalization protocols are based on wet chemistry strategies,^9−11^ such as the use of diazonium aromatic salt,^12^ click chemistry,^13^ alkylation,^14^ cycloaddition,^15^ or Diels–Alder reaction.^16^ In many cases, the obtained graphene suffers from non-controlled functionalization, lack of reproducibility, or even damages due to the aggressive treatments.^5^ Alternative strategies are being developed by physical methods in vacuum or ultrahigh-vacuum (UHV) conditions to functionalize substrate-supported graphene.^8,17^ These protocols are intrinsically clean and reproducible, showing negligible degradation of the graphene lattice.^8^ These approaches mostly rely on the controlled generation of atomic vacancies on graphene by low-energy ion irradiation of graphene substrates in UHV and their subsequent functionalization by neutral organic moieties.^8,18^ Alternatively, plasma-assisted (PA) dissociation of gases has been used to produce non-neutral species that can be linked directly to the carbon lattice.^17,19^ Unfortunately, UHV needs complex instrumentation, and their implementation in industry can be more difficult. Otherwise, PA dissociation has been limited to mono-atomic functionalization,^20^ but these limitations can be overcome if PA low-vacuum techniques are used to promote the dissociation of organic molecules in gas phase that immediately link to the graphene lattice. Thus, PA is a promising strategy for the covalent functionalization of graphene, as we address in this work. In addition, the PA low-vacuum process is fast, clean, cost-effective, and scalable.

Acrylate polymers are a type of significant vinyl polymers used in a wide range of industrial products, ranging from contact lenses or bone cement to cosmetics, coatings, adhesives orthopedics, textiles or drug administration. Acrylates in combination with other components (fibers, nanoparticles, and carbon materials) form interesting new hybrid materials for bioengineering. PHEA is a polymer broadly used as a hydrogel for lens, hemotherapy, in bioactive scaffolds, controlled release and applications that prove its biocompatible character.^21−28^ It is also a promising polymer with soft and hydrophilic properties and high water adsorption capacity,^29,30^ one of the key factors for biocompatible and bioengineering applications. Therefore, we expect this hybrid material to be a great candidate to study the modification of the graphene hydrophobicity and its improvement in the biocompatible behavior as in other cases where a polymer is combined with other materials.^21,28^

Here, we devise a new versatile two-stage functionalization approach to build large-area graphene–polymer hybrid nanoarchitectures as platforms with potential applications in biotechnology and sensing. In a first stage, the organic linker molecule *p*-aminophenol (*p*-AP) and the carrier H_2_ gas dissociate in a low-vacuum plasma chamber and the graphene layer is activated, enabling the covalent functionalization of the basal plane with the organic moiety. We functionalize the substrate-supported graphene with organic molecules in a low-vacuum clean plasma environment, as an unexplored alternative to the wet chemistry methods^5^ and UHV^8^ approaches. This (*p*-AP) organic molecule incorporates an end-group (phenol) which is intended to be the linker to further anchor more complex molecules and subsequently obtain a hybrid material. The key advantage of this strategy is the versatility of the method that enables to use specific linker molecules to functionalize graphene on demand.^18^ Recently, a similar platform has been demonstrated, especially suitable for high-performance bioelectronic devices.^31^

Afterwards, we carry out an in situ surface polymerization of 2-hydroxyethyl acrylate (HEA) monomer that grows in a controlled and reproducible manner^32^ from the OH group provided by the *p*-AP molecule, resulting in a robust and stable graphene/*p*-AP/PHEA hybrid functional material. Our method keeps the suitable properties of functionalized graphene, since no solvents or high temperatures that could lead to the material degradation are used enhancing, in contrast, the versatility of graphene.^21,33−36^ This approach is highly versatile and opens the door to the combination of a wide range of linkers and polymers with potential applications in several fields.^37−41^ For instance, graphene is a material with a large potential in high-performance humidity sensors, as reported in the literature.^42−44^ Therefore, the combination of the graphene sensitivity, the robustness of the covalent functionalization, and the water absorption capacity of the polymer make the hybrid platform proposed as a potential candidate to be an efficient humidity sensor. Accordingly, we show in this work a proof of concept of such application.

The topographic changes in graphene are sequentially characterized by atomic force microscopy (AFM), and the chemical functionalization is analyzed by X-ray spectroscopy (XPS) and Raman spectroscopy in both graphite surfaces and supported graphene samples. We ascertain that the graphene/*p*-AP/PHEA architecture is anchored covalently, in a stable and robust way, by sp^3^ hybridization of graphene bonds and controlled polymerization. Finally, we assess the sensitivity of our graphene–polymer hybrid platform developed by measuring the surface electrical properties by the four-point probe analysis after different relative humidity exposures as a proof of concept of a humidity sensor. This study describes a new versatile and reproducible route to design functional robust nanoarchitectures based on graphene that have shown breakthrough applications in different fields.

## 2 Experimental Section

### 2.1 Materials and Methods

#### 2.1.1 Graphene Functionalization

The first stage of covalent graphene functionalization is carried out by using an ASTEX AX 4500 electron cyclotron resonance (ECR) plasma-assisted equipment. A comprehensive description of the plasma system can be found elsewhere.^45^ During functionalization, the chamber pressure is 5.4 × 10^–2^ mbar, and the plasma power is 200 W. HOPG purchased from Tip Nano OÚ and Si/SiOx wafer-supported CVD graphene purchased from Graphenea Inc. are used as substrates. The *p*-aminophenol (*p*-AP) powder precursor is purchased from Sigma-Aldrich (H_2_NC_6_H_4_OH ≥ 98%), and H_2_ is used as a gas carrier. The second stage of functionalization and the final step to build up the hybrid material is carried out in an oven with a constant nitrogen flow. The monomer and initiator, 2-hydroxyethyl acrylate (HEA) (96%, contains 200–650 ppm of monomethyl ether hydroquinone as the inhibitor) and 2,2-azobis(isobutyronitrile) (AIBN), the reactants used to carry out in situ free-radical (FR) polymerization on the surface, are purchased and used as received from Sigma-Aldrich.

#### 2.1.2 Characterization

Room-temperature AFM measurements for topographical analysis are performed with two commercial equipment: (a) Nanoscope IIIa from Veeco (United States): Dynamic operation mode is selected, and silicon cantilevers (Bruker) with a nominal radius of curvature of 8 nm and nominal constant force of 1–5 N/m are used. (b) Instrument and software from Nanotec Electrónica S.L: Dynamic operation mode is employed, exciting the tip at its resonance frequency (∼75 kHz) to acquire topographic information of the samples. The structure of our graphene films is addressed by Raman spectroscopy using a confocal Raman microscope (S&I Monovista CRS+). Raman spectra have been obtained using a 532 nm excitation laser, a 100× objective lens (NA = 0.9), and an incident laser power of 6 mW. The chemical nature of the hybrid material is determined by XPS. XPS measurements are carried out under UHV conditions using a PHOIBOS 100 1D delay line detector electron/ion analyzer, monochromatic Al Kα anode (1486.6 eV), and pass energy of 30 eV.

#### 2.1.3 Proof-of-Concept Humidity Sensor

The water vapor absorption ability onto a sample surface because of the polymer presence allows using the hybrid material as a humidity sensor that is assessed at 0% relative humidity (RH), room RH (27 ± 3%), and top RH (94 ± 3%). The 0% RH data were acquired after the samples were placed 2 h in a vacuum environment (10^–6^ mbar). The 94 ± 3% RH data were acquired after 2 h of continuous exposure to a humidity-saturated environment using a wet paper in an airtight Petri dish (see Figure S5 in Supporting Information). The room RH (27 ± 3%) corresponds to the laboratory ambient conditions. Measurements correspond to *I*–*V* curves in a four-point probe system JANDEL RMS2 Universal Probe with continuous current (from 0.1 to 10 μA). We apply the standard equation *R*_□_ = 4.53·Δ*V*/*I* for sheet resistance (*R*_□_) calculations (Δ*V* being the change in voltage measured between the inner probes and *I* the current applied between the outer probes).

### 2.2 Build-Up Protocol for a Graphene–Polymer Hybrid Functional Material

The build-up strategy includes two main stages: the linker attachment consisting of a covalent functionalization with the organic molecule *p*-aminophenol (*p*-AP) and the 2-hydroxyethyl acrylate (HEA) in situ polymerization on the surface.

#### 2.2.1 Linker Covalent Functionalization

**Figure 1:**
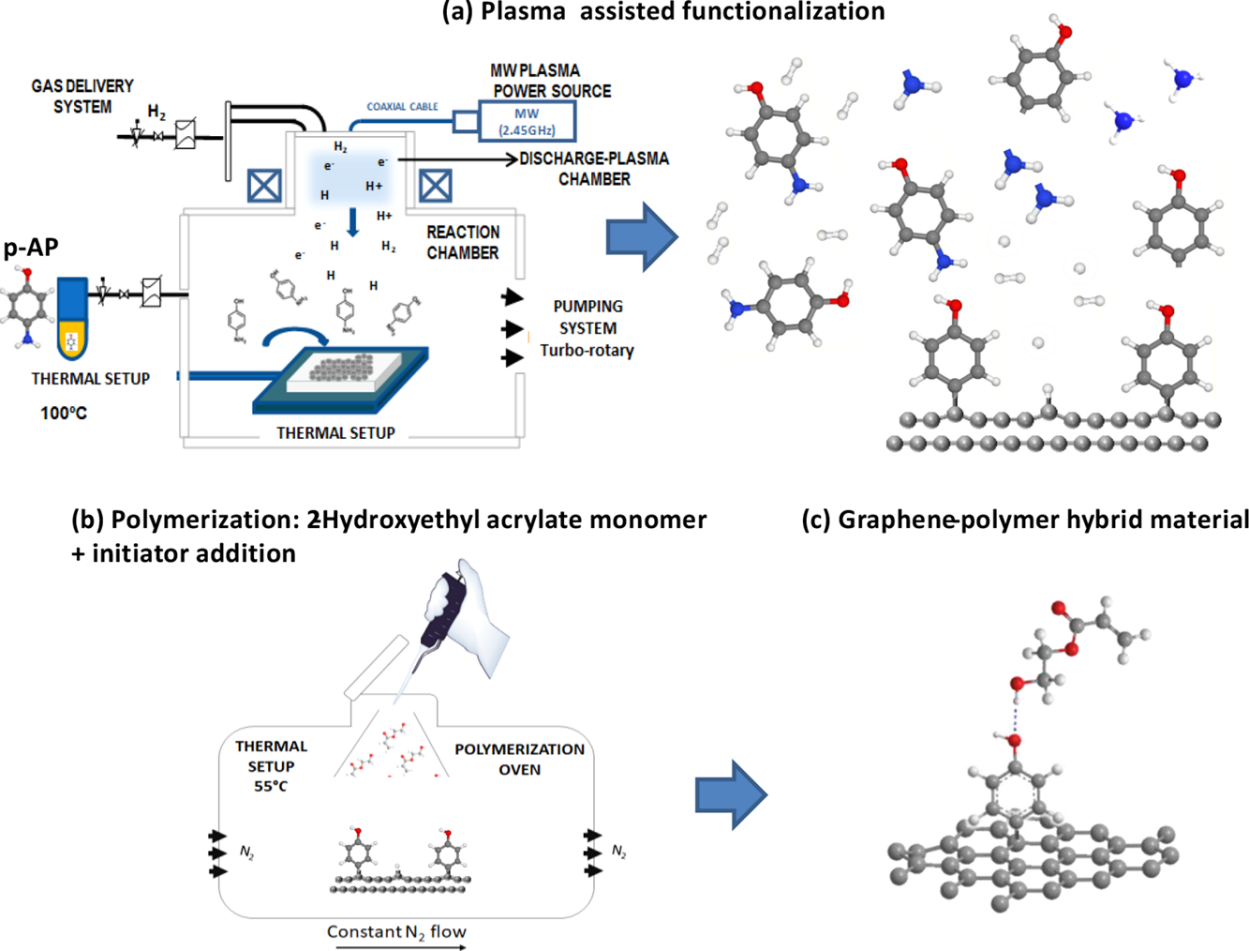
Schematic illustration of the synthesis of a polymer–graphene hybrid functional material: (a) first stage: dissociation of H_2_ and *p*-AP linker in the plasma phase, soft hydrogenation of graphene lattice and functionalization with aromatic groups containing OH moieties. Graphene hydrogenation and linker attachment occur sequentially. (b) Functional molecule attachment of the HEA monomer by in situ polymerization. Scheme (c) represents the anchoring of a monomeric unit to the phenolic linker as a representation of the beginning of the polymerization process to obtain our final hybrid platform purposed.

Figure 1a depicts the first step related to the plasma functionalization process carried out in a low vacuum. The *p*-AP precursor powder is evaporated into the chamber at 100 °C during 10 min, while the graphene or HOPG samples are at room temperature. After the *p*-AP gas pressure is stabilized, we introduce the H_2_ gas carrier and switch on the plasma during 30 s (see Figure S1 from the Supporting Information to find the study for the plasma time selection). The plasma activation dissociates atomic H and the organic precursor in radicals, as depicted in Figure 1a, right side. Atomic H produces a graphene buckling effect by side hydrogenation, resulting in graphene-enhanced reactivity due to the sp^3^ soft hybridization of the basal plane.^46,47^ Note that this ECR plasma produces around 5–10% atomic H. This process enables the attachment of the *p*-AP organic radicals previously activated by plasma. In this way, a high density of functionalization can be achieved, controlling time and dose (see Figure S1). One plausible reaction path is the plasma-assisted dehydrogenation^48^ or dissociation^49^ of the amino group in plasma, leaving behind aromatic groups with hydroxide moieties that anchor to the buckled graphene lattice, as pointed out by our XPS results below. We also tested the attachment of the molecule by carrying out an ethanol wash and taking AFM images before and after the process. We observe that the molecule remains intact (all AFM images shown in this work were measured after this cleaning step). As a result, the phenol end-group becomes the linking moiety to anchor more complex molecules.

#### 2.2.2 Polymer–Graphene Hybrid Material

Figure 1b depicts the second stage that consists of controlled in situ polymerization on the covalent functionalized graphene surface. The monomer mixture is prepared and stirred in a round-bottom flask with 1 mL of HEA monomer mixture +0.5% v/v AIBN. We set the reaction temperature at 55 °C and at a constant N_2_ flow in order to perform a slow and controlled polymerization based on previous kinetic studies reported in the bibliography.^29,32^ We are interested in a slow polymerization rate to obtain a low surface coverage. In this work, two in situ polymerizations are carried out where one sample aims to have the lowest amount of polymer on the surface (L-PHEA) and the other intends to achieve higher polymer coverage on graphene (H-PHEA). For L-PHEA, the reaction time is 30 min, while for H-PHEA, it is 1 h, and in addition, the polymerization process was repeated once.

The process of anchoring the first monomer unit to our functionalized graphene surface proceeds as follows. Initially, as the temperature increases, the initiator (AIBN) fragments into primary radicals by the cleavage of weak bonds. These radicals find a monomer molecule to react with their double bond and start the reaction. During polymerization, several monomer molecules are added from the cleaved double bond, thus forming macroradical chains attached to this first monomer represented in Figure 1c. In addition, and simultaneously, the presence of hydroxyl groups on our surface results in the formation of hydrogen bonds between the growing polymer and the *p*-AP linker molecule anchored on the graphene surface. Similar processes have been studied by the radical polymerization of acrylate monomers, such as poly(hydroxyethyl methacrylate), PHEMA, or poly(butyl methacrylate), PBMA, to obtain nanocomposites based on the reaction of the monomer with nanosilica or by the addition of graphene oxide (GO).^50−52^ This reaction is selective and requires the presence of oxygen-containing groups in order to accomplish the attachment to the acrylate and, therefore, can be a marker of the presence of free −OH groups in the linked *p*-AP. The resulting graphene–polymer samples are washed using ethanol to remove the possible physisorbed monomer and polymer (all AFM images shown in this work were measured after this cleaning step). The final anchoring of the monomer to the linker molecule covalently bonded on the graphene surface is depicted in Figure 1c, thus showing the first monomeric unit anchoring to the phenolic linker as a representation of the beginning of the polymerization process to obtain our final hybrid platform purposed. HOPG samples are used for the surface investigation and validation of the protocol due to their extreme flatness compared to the as-received commercial graphene ones. In the following sections, we also validate the functionalization protocol with CVD graphene samples on Si/SiO_*x*_ substrates.

## 3 Results and Discussion

### 3.1 Morphological Characterization of the Different Stages in the Synthesis of a Polymer–Graphite Hybrid Material

**Figure 2:**
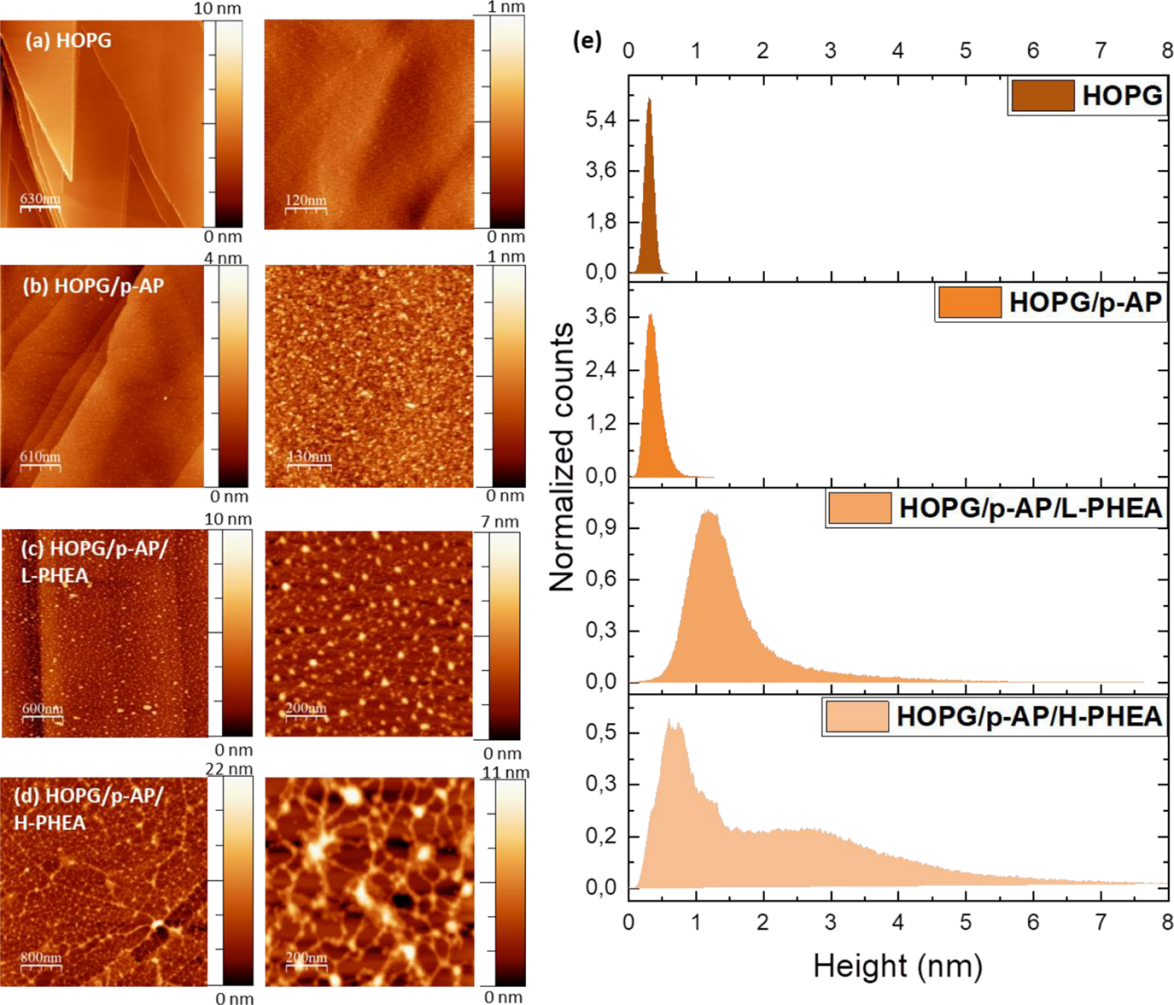
(a–d) AFM overview images (left) and their respective zooms (right) of HOPG after each functionalization stage. (a) Pristine HOPG. (b) Functionalized HOPG with *p*-AP molecules. (c) Functionalized HOPG with *p*-AP molecules and polymer during 30 min. (d) functionalized HOPG with *p*-AP molecules and polymer during 1 h. (e) Evolution of the height values in every step obtained from AFM images.

Two types of samples were polymerized in situ with HEA, one with a low amount of polymer on the surface (HOPG/*p*-AP/L-PHEA) and another with a higher polymer coverage (HOPG/*p*-AP/H-PHEA). Figure 2a–d shows AFM images recorded after every step in the functionalization process. The morphology evolution during the different stages is summarized in Figure 2e that shows the height distribution of characteristic images for each case and supports the description of the images in the text below.

Figure 2a shows a pristine HOPG surface used as a reference of the initial surface before experiments. The surface shows the characteristic terraced and stepped morphology. The higher resolution image at the right was taken on one of the terraces showing a featureless morphology, with the root-mean-square, rms, or surface roughness, smaller than 0.03 nm. Figure 2b shows the surface morphology after the first stage of covalent functionalization with the linker molecule, *p*-AP. The stepped morphology is still observed now, and the surface has been altered due to the surface buckling and *p*-AP adsorption induced by the hydrogenated plasma, which results in the modification of the surface roughness of pristine HOPG increasing by an order of magnitude around 0.12 nm. The HOPG and HOPG/*p*-AP samples show a narrow peak, indicating that most surface locations have a similar height (0.33 nm). For the HOPG/*p*-AP sample, this peak is wider and shifted to slightly higher values because of the surface roughening and the *p*-AP molecules. The roughening as well as the different morphology allows us to confirm the incorporation of *p*-AP molecules onto the surface. Figure 2c,d and the corresponding zoomed images show the morphology of the sample after in situ PHEA polymerization for the lowest (HOPG/*p*-AP/L-PHEA) and highest (HOPG/*p*-AP/H-PHEA) polymer coverage on graphene, respectively. The wide images in Figure 2c,d indeed show the terraced and stepped morphologies of the graphite substrate. A closer inspection of the morphology on the terraces reveals a clear change. Thus, in the case of HOPG/*p*-AP/L-PHEA, a surface coverage close to 20% and a surface density of about 350 structures/μm^2^ are observed. A high density of features, with heights between 0.3 nm and 4.6 nm is found, a majority of them being of globular morphology with an average height around 1.2 nm. These changes confirm that polymerization has taken place and that it is stable as the samples were measured after being washed with water. Finally, for the HOPG/*p*-AP/H-PHEA sample, the large image in Figure 2d and in more detail its corresponding zoom show globular structures similar to the previous ones and a new network formation, with a variation of surface heights in the range of 0.1–8 nm and reaching a surface coverage slightly higher than 50%. The globular-shaped protuberances can be attributed to the clustering of events of heights larger than 1.5 nm, showing a longer tail for large height values because of the scarce higher protuberances. Most remarkable, however, is the formation of a network of structures on the substrate, which is due to enhanced two-dimensional polymerization on the functionalized surface, seeing how the peak shifts to slightly lower values and exhibiting a height range between 0.9 and 1.5 nm. According to the experimental process described in Section 2.2, a first monomer anchors on the *p*-AP molecule through the hydroxyl group, and then from there the polymer grows. Larger aggregates represent a larger polymer chain. In addition to the domains observed as aggregates in Figure 2c,d, interconnections between polymer chains are also observed, forming a surface network that increases with the polymer coating. The coverage found on the surface is homogeneous in both cases, being higher for the polymerization with H-PHEA, as expected. These results point out a highly controlled and homogeneous polymerization method in the functionalized graphene (not in a stacked manner), where the degree of surface coating according to the future application of the hybrid platform can be tailored à-la-carte. It is noteworthy that the polymer structures on the graphene surface remain unaltered after repeated surface washings as well as during sample measurement, evidencing a robust attachment of the polymer to the graphene-functionalized surface.

### 3.2 Chemical Characterization of the Different Stages in the Synthesis of a Polymer–Graphite Hybrid Material

**Figure 3:**
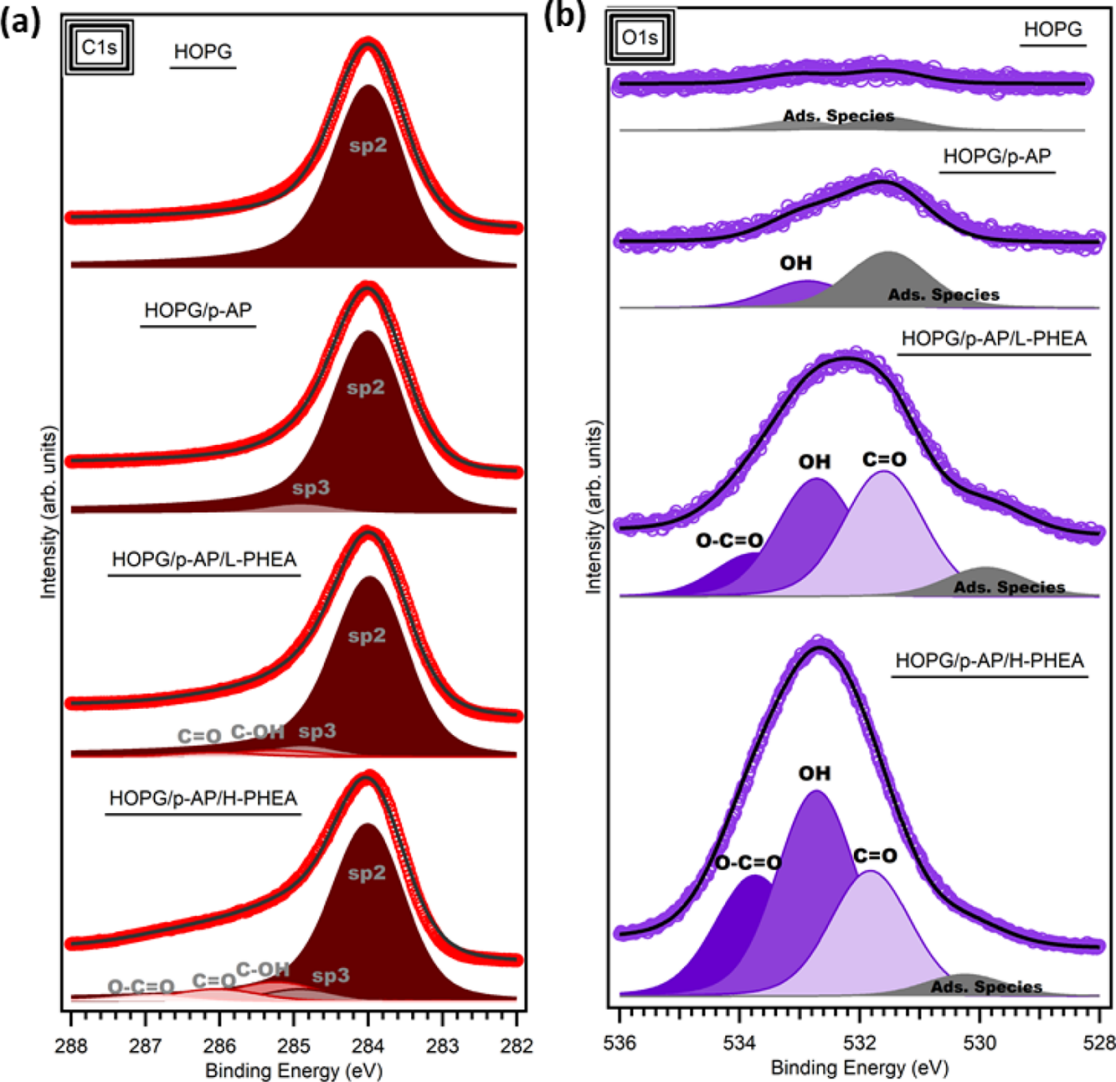
Core-level evolution of (a) C 1s and (b) O 1s. From top to bottom: pristine HOPG, HOPG sample after *p*-AP functionalization (HOPG/*p*-AP), and HOPG samples after the two polymerization processes (HOPG/*p*-AP/L-PHEA and HOPG/*p*-AP/H-PHEA).

The chemical nature of the hybrid functional material has been characterized by XPS. Freshly cleaved HOPG is used as a reference substrate to study the chemical variations induced in the consecutive stages, the *p*-AP-graphite and PHEA-p-AP-graphite architecture. Figure 3a,b shows the C 1s and O 1s core-level spectra evolution, respectively, obtained from the samples after each stage; from top to bottom: pristine HOPG, HOPG sample after *p*-AP functionalization (HOPG/*p*-AP), and HOPG samples after the two polymerization processes (HOPG/*p*-AP/L-PHEA and HOPG/*p*-AP/H-PHEA). The C 1s core level (Figure 3a, upper spectrum) of the pristine HOPG shows a single component at 284.0 eV that can be assigned to the sp^2^ lattice of graphite.^53−55^ After *p*-AP functionalization, a new component at 284.9 eV is observed. This one is ascribed to the sp^3^ hybridization of graphite carbon with low intensity in comparison with the sp^2^ signal, as expected, considering the contribution of the bulk HOPG.^52^ In principle, this signal could be related to the bonding with organic groups from the *p*-AP precursor or atomic hydrogen (H). We also check that the main sp^2^ component remains almost unchanged, confirming that no damage was induced in the sample by plasma-assisted processing. After the polymerization, new contributions appear at 285.2 and 286.0 eV, which are ascribed to C–OH and C=O, respectively, from the hydroxyls and carboxylic groups from PHEA. Finally, relative to the last C 1s spectrum of Figure 3a, the HOPG/*p*-AP/H-PHEA sample reveals the increment of polymer conversion on the graphite surface with the enhancement of the contributions at 285.2, 286.0, and 286.8 eV, assigned to the oxygen bonds, C–OH, C=O, and O–C=O respectively, from the hydroxyls and carboxylic groups from the PHEA structure.^56−58^ This signal enhancement is a direct consequence of the increase in the polymer coverage, in good agreement with the previous morphology seen in Figure 2d. Figure 3b shows the evolution of the O 1s core-level spectra obtained after each stage.

For the pristine sample, the spectrum presents two small components that can be assigned to the environmental adsorbed species as a consequence of the exfoliation in air.^52^ The next spectrum corresponds to the *p*-AP covalent functionalized sample, HOPG/*p*-AP, where, in addition to the adsorbed species, a new component at 532.8 eV appears. We ascribe this new component to the OH groups of the *p*-AP molecule, confirming its presence.^48,49,59,60^ We also see the increase of adsorbed species, which is easy to explain considering the plasma activation of the HOPG surface and exposure to ambient air before the XPS analysis. This activation enhances the adsorption of molecules as the sample surface is not completely saturated during the covalent functionalization experiment. This intentioned saturation control allows us to maintain the proper electronic properties of graphene, as we see below (see Figure S1 in the Supporting Information). Regarding the functionalized surface after the polymerization reaction, as expected for a PHEA coverage, there are multiple components in the O 1s peak: 531.6, 532.8, and 533.7 eV, assigned to the carbonyl (C=O), hydroxyl (OH), and ester (O–C=O) species, respectively.^56,57,61^ Two similar spectra are observed in both L-PHEA and H-PHEA polymerizations, with the only difference in the intensity of each characteristic contribution being larger after H-PHEA polymerization, as expected. We also observe a small remaining contribution due to the adsorbed species at 529.9 eV that may be a slight surface oxidation due to the existence of steps, defects, grain boundaries or exposed atomic sites after repeated surface washings.

### 3.3 Graphene/Polymer Functional Hybrid Material for Humidity Sensors

The study from HOPG has allowed us to develop, characterize and validate the efficiency of a two-stage graphene functionalization process based on a linker and functional molecule attachment on the surface to obtain a graphene–polymer hybrid platform. However, from the large-scale application point of view, the relevant next step consists of the transfer of this protocol to a graphene sample. In this scenario, the substrate morphology and the structure of functionalized graphene samples have been sequentially characterized by AFM and Raman spectroscopy in every step of the process (see Figures S1 and S2 in the Supporting Information). The analyses confirm, overall, that the functionalization process takes place similarly on graphene as remarkable differences in the final morphology of the samples or in the chemical mechanism involved are not observed. We conclude that the synthesis of a polymer/graphene hybrid functional material successfully works, which opens the door to its application in devices.

**Figure 4:**
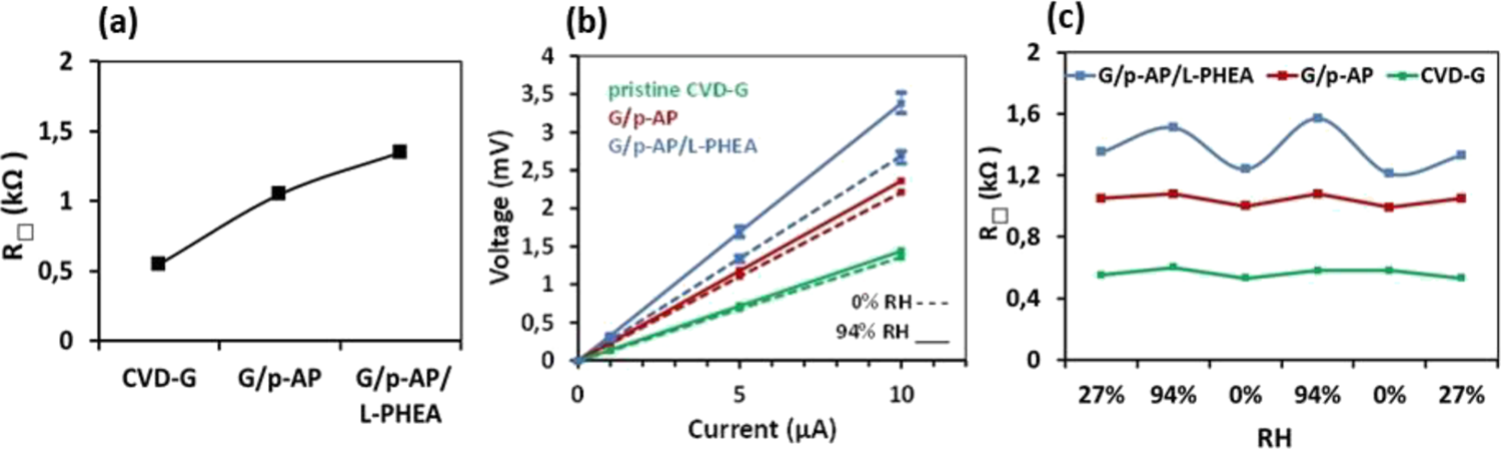
Initial studies about the hybrid material as a humidity sensor comparing pristine graphene, G/*p*-AP, and G/*p*-AP/L-PHEA. (a) Average sheet resistance of the samples. (b) *I*–*V* curves of the samples at 0 and 94% RH. (c) Dynamic response of the samples exposed to humidity.

One of the main challenges of covalent graphene functionalization is to retain suitable electrical properties of the graphene layer after processing. In order to study the changes in the conductivity of graphene throughout the different stages, we have measured systematically before and after each stage the sheet resistance (*R*_□_) of the layer by means of a four-point probe analysis at room temperature. Figure 4a shows the *R*_□_ variation of the samples, extracted from the analysis of the slope of the corresponding *I*–*V* curves performed at several points of the samples under room conditions (see Materials and Methods). The initial pristine graphene layer exhibits an average sheet resistance value of 0.55 kΩ after the cleaning procedure that we apply to our samples, 2 h at 340 °C, in a high vacuum (see Figures S3 and S4 in the Supporting Information), being a standard result due to the high quality of the material. This resistance value increases to 1.05 kΩ on average after the functionalization with *p*-AP, as expected, considering the induced sp^3^ hybridization of the graphene lattice. As mentioned above, the functionalization density, and thus the hybridization degree, obviously depends on the plasma-assisted processing time, 30 s, as this parameter determines the precursor dose. The processing time used is selected after studying its influence on the electrical properties and the structure of graphene as a balance between the functionalization density desired and the reduction of conductivity allowed for the intended application (Figure S1 in the Supporting Information). After the polymerization step, the sheet resistance increases, up to 1.35 kΩ, as seen in the last point of Figure 4a, which is a reasonable value for further applications. The hydrophilic behavior of the polymer and its water absorption ability allow the use of this hybrid material as a potential humidity sensor. Accordingly, we test it by measuring the electric response from different humidity conditions. We then evaluate the influence of water absorption and release and both the static (fixed RH) and dynamic (changing RH) conditions. Thus, we have analyzed the resistance evolution as a function of RH in three different scenarios: 0% RH, room RH (27 ± 3%), and top RH (94 ± 3%) at each stage of the process. Figure 4b presents the corresponding *I*–*V* curves measured on each sample. Finally, Figure 4c presents the dynamic response of our platform as a sensor, applying different cycles of humidity conditions. Figure 4b shows that the pristine graphene, in green, has a low sensitivity to humidity variations, as the signals that correspond to 0% RH and top 94% RH nearly overlap. This result is in good agreement with the previous studies of monolayer graphene resistive humidity sensors, as small resistance changes have been usually observed.^43,62−64^ A similar behavior is observed when p-AP is covalently bonded to graphene, G/*p*-AP in red, which does show a small increase in resistivity as the humidity increases, in agreement with previous reports on this effect as a function of graphene doping^65^ and band gap opening.^66^

However, when the polymer is anchored to the surface, the sensitivity of this graphene-based hybrid platform, in blue, becomes outstanding. This is related to the hydrophilic character of the polymer and its ability to absorb water into its structure.^30^ The resistance increase after water absorption is attributed to the changes in graphene doping. The Raman analysis of the pristine graphene (see Figure S2d in the Supporting Information) shows that the 2D peak position is around 2682 cm^–1^ pointing out toward n-doping.^67,68^ The water absorption is known to induce p-doping in graphene, as water molecules behave as electron acceptors^42,43,69^ when directly into contact with the graphene surface. As the Raman 2D peak of the functionalized samples is in the same position as that of pristine graphene in Figure S2d in our samples, the water-induced charge transfer decreases the graphene n-doping, increasing its resistivity.

Regarding the reproducibility and sensitivity of the materials as a sensing platform, Figure 4c shows again that the samples of graphene and graphene covalently functionalized with *p*-AP exhibit a low interaction with the dynamic humidity change in the environment (red and green lines). However, a remarkable improvement in the detection performance of the G/*p*-AP/L-PHEA material is observed due to the natural adsorption and release of water molecules on the polymer (blue line). From a quantitative point of view, the resistance variations or sensitivity, *S*_R_, exceed 29% (*R*_□_ (94%RH) = 1.57 kΩ, *R*_□_ (0%RH) = 1.21 kΩ, from Figure 4c; see definition of *S*_R_ in Table S1). Table S1 compiles a performance comparison of this work with a number of resistive humidity sensors reported in the literature^44,70^ that confirms the high sensitivity of the hybrid material to the humidity conditions. It is thus demonstrated that chemical modification by poly-2-hydroxyethyl acrylate on the surface is a feasible way to improve the sensitivity of graphene to humidity.

## 4 Conclusions

In summary, we present a novel route to synthesize a polymer/graphene hybrid functional material that consists of a two-stage process. In the first stage, a low-vacuum hydrogen-assisted plasma activation process produces a stable and covalent graphene-functionalized platform with a small linker molecule (*p*-AP). This unexplored physical protocol is clean, reproducible, easily scalable, and cost-effective. The second stage consists of in situ radical polymerization on the functionalized graphene surface, leading to a PHEA/graphene hybrid material. This strategy is highly versatile and opens the door for the design of a wide range of graphene–polymer or biopolymer hybrid platforms for biosensing devices. AFM, XPS, Raman, and four-point probe sheet resistance analyses confirm that the graphene/*p*-AP/PHEA architecture is covalently anchored by sp^3^ hybridization of graphene, retaining suitable electronic properties after the polymerization process. We have successfully tested the hybrid material as a resistive humidity sensor since the hydrophilic PHEA polymer modifies the hydrophobicity of graphene, finding an outstanding sensitivity to humidity variations, *S*_R_ = 29%, competing with previous sensors based on graphene, graphene oxide, reduced graphene oxide, or polymer-functionalized graphene. In addition, this proposed graphene–polymer hybrid functional material leads the way for further biotechnological applications that will be studied in future research projects in order to expand its field of potential applications.

## Supporting Information Available

Processing time study for the plasma-assisted functionalization of graphene, including AFM, Raman, and four-point probe analyses, and its effects on the density of defects; application of functionalization protocol to graphene samples including AFM and Raman characterization; AFM and Raman characterization of transferred graphene cleaning protocol; experimental setup description for saturated humidity measurement; and comparison of the humidity sensor sensitivity results obtained with the state-of-the-art resistive sensor based on graphene (PDF)
