# Supplementary material for: Acrylates Polymerization on Covalent Plasma-Assisted Functionalized Graphene: A Route to Synthesize Hybrid Functional Materials
Source: ACS Appl Mater Interfaces. 2023 Sep 22;15(39):46171–80. doi: 10.1021/acsami.3c07200 (PMC10561134; doi:10.1021/acsami.3c07200)
Supplement: Supplementary file 1 — am3c07200_si_001.pdf [file am3c07200_si_001.pdf]

Supporting Information

Acrylates polymerization on covalent plasma assisted functionalized graphene: a route to synthesize hybrid functional materials

Roberto Muñoz[a*+], Laia León-Boigues[a,b*+], Elena López-Elvira[a], Carmen Munuera[a], Luis Vázquez[a], Federico Mompeán[a], José Ángel Martín-Gago[a], Irene Palacio[a*], Mar García-Hernández[a]

[a] Instituto de Ciencia de Materiales de Madrid, (ICMM-CSIC), Sor Juana Inés de la Cruz 3, E-28049, Madrid, Spain.
[b] Universidad Complutense de Madrid, 28040 Madrid, Spain

*e-mail: rmunoz@icmm.csic.es; laleboi@ictp.csic.es; i.palacio@csic.es
[+] These authors contributed equally

INDEX

1. **Plasma-assisted functionalization of graphene: Processing time study**
2. **Graphene/polymer functional hybrid material**
3. **Graphene cleaning protocol. AFM and Raman characterization**
4. **Top humidity measurement set-up. Resistive humidity sensors performance**

1. Plasma-assisted functionalization of graphene: Processing time study

The functionalization density depends on the plasma assisted processing time, as this parameter determines the precursor dose. In fact, this time is proportional to the amount of linker molecules attached covalently to graphene. As the covalent bonds induce a sp^3 hybridization suspected to lower the electrical performance of the samples, accordingly, the processing time influences the electrical properties of graphene and it must be selected as a balance between the functionalization density desired and the reduction of conductivity allowed for the intended application. In Figure S1 we show the characterization results of different samples processed and functionalized as a function of plasma assisted processing time (pristine graphene, and graphene/p-AP with 30” and 60” of functionalization). We have analyzed the samples by AFM, Raman spectroscopy and four-point probe analysis. The morphological analysis by AFM in figure S1 (a-c) does not show relevant changes as a function of the functionalization time due to the intrinsic roughness of the graphene transferred to the SiOx substrate. However, the Raman spectra in figure S1 (d) show great changes in the structure of the graphene films as a function of functionalization time. We clearly see that the D peak attributed to sp^3 defects, emerges for 30” and it is enhanced with 60”. In figure S1(e) we compare the ID/IG and I2D/IG intensity ratios as a function of plasma assisted processing time. It can be observed that the ID/IG ratio increase is almost linear as a function of processing time indicating a linear relation between the amount of sp^3 defects and processing time. Also, de I2D/IG ratio decreases as an indication of the higher number of defects. Finally, we measure the sheet resistance of the same samples as a function of processing time. We see that the linear ratio disappears. The sheet resistance increases slightly for 30” of functionalization but there exists a dramatic increase of the sheet resistance at 60” processing time that is not reasonable for its application. We conclude that the final resistance value for 30” is more suitable to continue with the second step of functionalization.

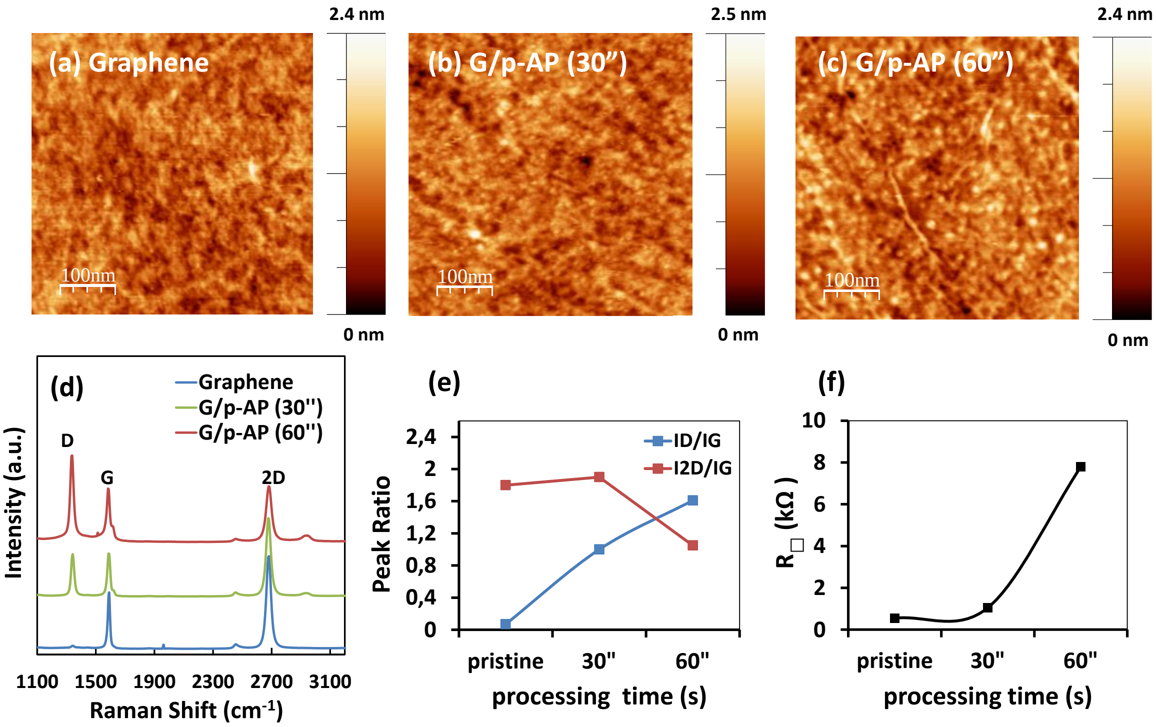

Figure S1. Qualitative study of the density of sp^3 defects and its effect in the morphology, chemical structure and electrical conductivity of graphene. (a-c) CVD graphene morphological evolution after plasma assisted functionalization as a function of time. No relevant changes are observed due to the intrinsic roughness of the graphene transferred to SiOx (a) Pristine graphene sample after thermal annealing at 340ºC, 2h. (b) Pristine graphene + p-aminophenol, 30" processing time. (c) Pristine graphene + p-aminophenol 60" processing time. (d) Raman spectroscopy evolution of CVD graphene samples after plasma assisted functionalization with p-AP as a function of time. (e) Peak ratio analysis of the spectra in (d). The sp^3 hybridization increase sharply if we apply more than 30" of plasma. (f) Sheet resistance analysis of CVD graphene samples after plasma assisted functionalization as a function of time. It is clearly observed the enhancement of sheet resistance from pristine sample as a function of time. We can see that if we apply more than 30" of plasma the sheet resistance increases sharply.

2. Graphene/polymer functional hybrid material

Figure S2 (a-c) shows the topography images of a pristine CVD graphene on Si/SiO_x and its evolution after each stage, confirming the functionalization process by clear topographic changes in exposed graphene surface. Figure S2(d) shows the corresponding Raman spectra of graphene (black spectra), with functionalization (blue spectra) and after polymerization stage (red spectra). The evolution of the process can be analysed quantitatively by studying the different heights and rms obtained from AFM analysis, as we did previously with HOPG in the main text. In the case of graphene, the morphology is prone to be influenced by the intrinsic roughness of the substrate, Si-SiO_x here. This is why the values of the root mean square, rms, or surface roughness, from figure S2(a) (0.291 nm) start out higher than those observed previously at figure 2 in the main text for freshly cleaved HOPG (0.03 nm), as expected. It should be noted that the graphene samples used in this study were previously annealed under vacuum to remove residues from the inevitable transfer process (see figures S3 and S4 below). After the first covalent functionalization with p-AP on graphene G/p-AP, the AFM image in figure S2(b) does not show a distinguishable difference in rms, 0.293nm (0.12 nm in the main text for HOPG/p-AP). This is due to the low size of the molecule and the mentioned starting roughness. This fact justifies even more the utilization of freshly cleaved HOPG single crystals to evidence the process in our preliminary fundamental approach. However, after anchoring with the L-PHEA, appreciable surface changes are observed in figure S2(c). As previously shown in this work (figure 2c in the main text), higher domains corresponding to the polymer presence are found being homogeneously distributed over the entire surface. In this case the height reveals the anchoring of the polymer to the monolayer graphene surface, showing an average height of 1.84 nm after polymerization stage, in the same order of the polymer-HOPG samples, considering the substrate induced roughness to graphene surface. We point out here that Raman analysis attempted on HOPG is not useful to assess the structure of the functionalized samples as the contribution of the molecules is not detectable in the spectrum due to the high response of graphite related peaks (not shown). However, Raman spectroscopy analysis of graphene on Si/SiO_x substrates is useful for the purpose and is therefore shown here. We see in the spectrum of monolayer graphene, figure S2(d) black-line, the contribution from the G peak at 1586 cm^{-1} and 2D peak around 2682 cm^{-1} [1]. We find that after the first stage

of functionalization the defect related D peak at 1342 cm^{-1} emerges, attributed to the sp^3 hybridization of the carbon lattice [1-4] by plasma functionalization as discussed in the main text during the XPS analysis. As the functionalization proceeds at room temperature the etching of the graphene layer is not the prevailing process while the covalent attachment of the plasma species is favoured by the simultaneous buckling [3] and intrinsic roughness of supported graphene. Finally, relative to the polymerization stage, red spectrum in figure S2(d), it can be concluded that the polymerization reaction does not notably influence the number of defects or sp^3 character of the carbon lattice and it is produced as in HOPG samples.

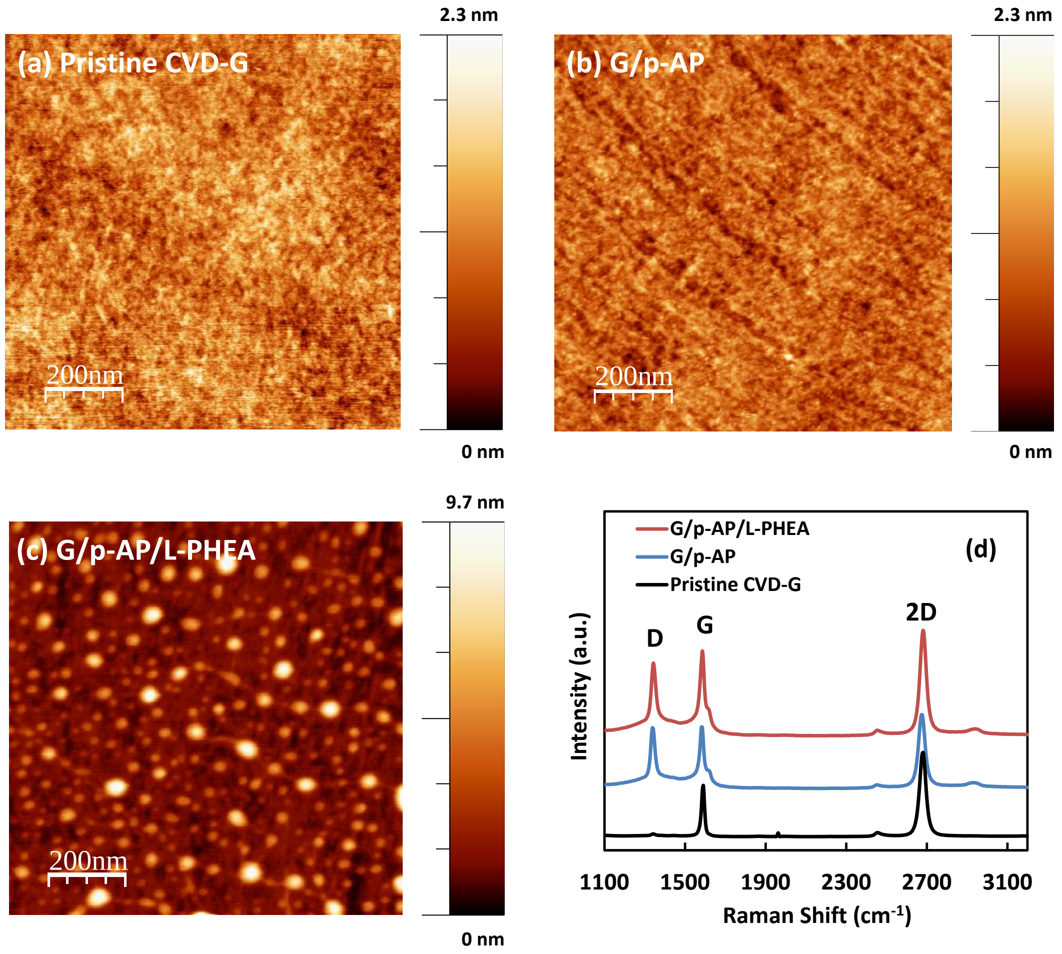

Figure S2. Functionalization protocol on supported graphene. (a-c) AFM topographic images of silicon oxide supported CVD graphene before and after each functionalization stage. a) Pristine graphene; b) pristine graphene after p-AP functionalization (G/p-AP); (c) Graphene after the polymerization process (G/p-AP/L-PHEA). (d) Raman spectra of the corresponding samples.

3. Graphene cleaning protocol AFM and Raman characterization

The commercial graphene surface can be contaminated with polymer residues as received. We systematically apply to the graphene samples a cleaning protocol based on thermal annealing in high vacuum, before functionalization. We selected the annealing parameters, T and time, after, comprehensive characterization of the roughness by AFM and structure by Raman.

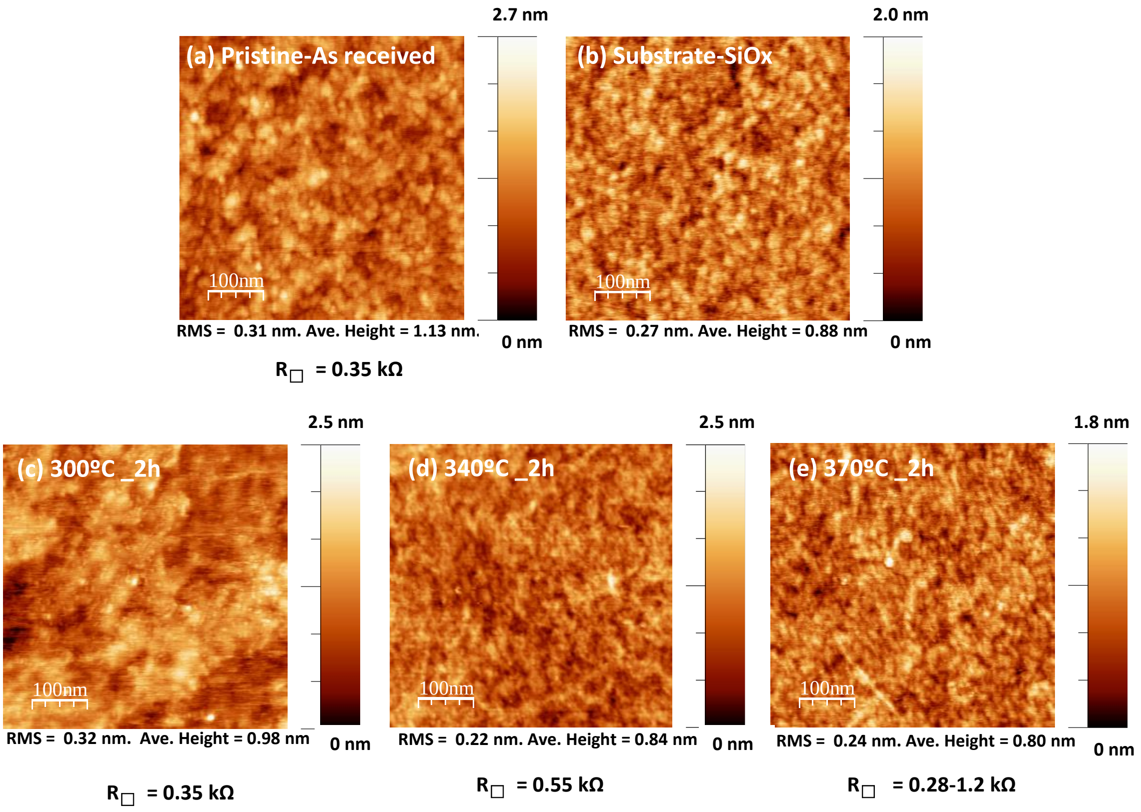

Figure S3. Graphene CVD cleaning protocol before processing. The evolution of the roughness parameters RMS and Average Height and the sheet resistance are observed under the images. (a) AFM topographic image of Pristine- as received sample. (b) AFM topographic image of the SiO_x substrate. AFM topographic images of the sample after thermal annealing 300ºC, 2h (c); 340ºC, 2h (d); 370ºC, 2h (e). We observe that the RMS and average height parameters are higher in as received graphene (a) comparing with the supporting substrate in (b), this related to the polymer residues expected. After first annealing at 300ºC, there is no evident changes. After annealing at 340ºC in (d) we see that the roughness parameters decrease to values comparable to the supporting substrate. This means that the polymer residues have mostly disappeared. The sheet resistance increases slightly. Finally, if we apply an annealing of 370ºC the roughness parameters maintain their values but the sheet resistance has a high dispersion. See in the next image the corresponding Raman analysis.

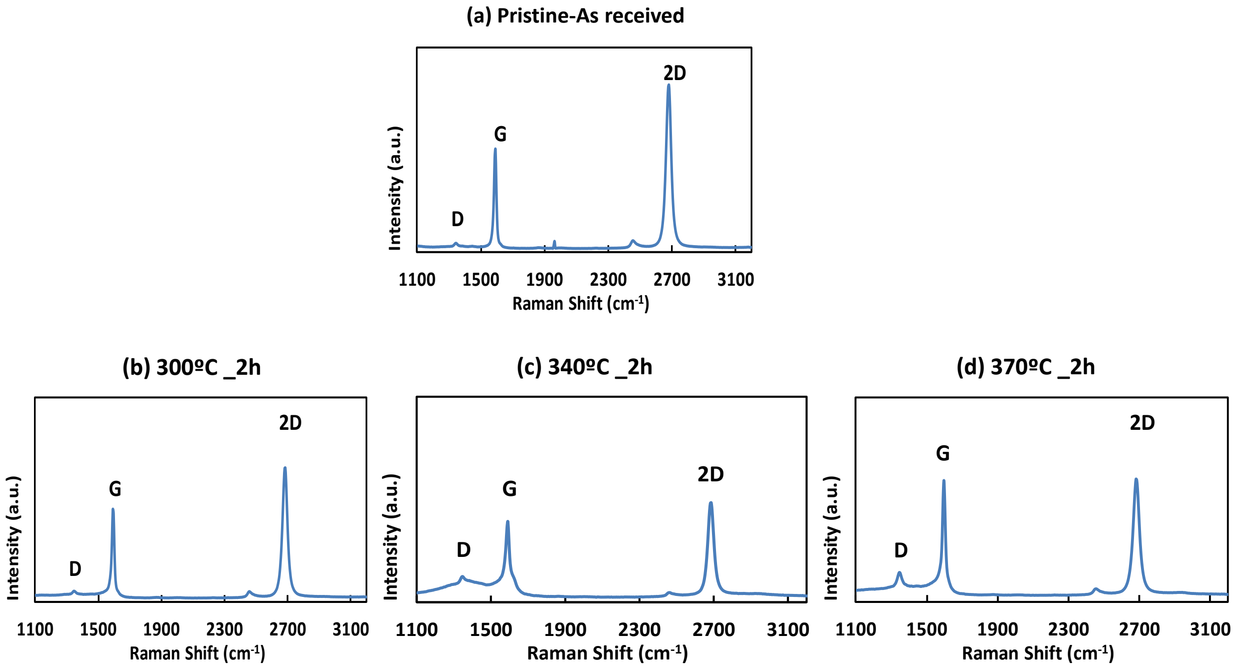

Figure S4. Graphene CVD cleaning protocol before processing. Evolution of the Raman spectra as a function of annealing temperature. (a) Pristine- as received sample. (b) Sample after thermal annealing 300ºC, 2h; (c) 340ºC, 2h.; (d) 370ºC, 2h. We observe that if we apply an annealing of 370ºC the D peak emerges clearly in many points. The final applied annealing in the main text is 340ºC, 2h.

4. Top humidity measurement set-up. Resistive humidity sensors performance

The response of the samples to top humidity (94±3% RH data) was acquired after 2 hours of continuous exposure to humidity-saturated environment using a wet paper inside an airtight petri dish, Figure S5.

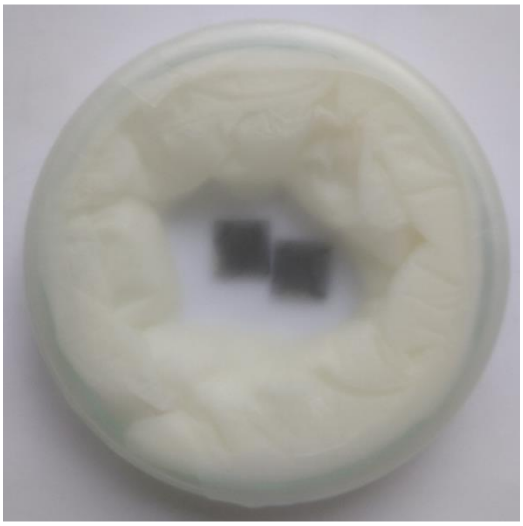

Figure S5. Experimental set-up for saturate humidity measurement top saturated humidity (94±3%), composed by a parafilm sealed petri dish with wet paper. The photograph shows two samples, pristine graphene and graphene after polymerization. The photograph is blurry due to the humidity saturation on the glass.

Table S1. Performance comparison of different resistive humidity sensors based on graphene, graphene oxide (GO), reduced graphene oxide (r-GO) and functionalized graphene with polymers.

**Material**	**Preparation Method**	**Operating Temperature**	**Sensitivity S_R**	**Sensitivity S_H**	**Humidity Range**	**Ref.**
Graphene	CVD	R.T.	---	0,31%RH	1-96%	5
Multilayer Graphene	CVD	25ºC	10-17%	---	15-80%	6
Double-layer graphene	CVD	25ºC	0.933%	--	20-100%	7
Wrinckled Graphene	CVD	25%	3% (graphic data)	--	11-95%	8
r-GO	Rapid thermal annealing	25%	0,075-35,3%	--	20-95	9
Reduced Graphene oxide (r-GO)	Chemical reduction	25%	17.6%	--	10-70%	10
N-doped r-GO fiber	Thermal annealing	R.T.	0,32-4.51%	--	6.1-99,9%	11
r-GO	Exfoliation at liquid/air interface	R.T.	6%	--	4.3-75,7%	12
HFBI-r-GO	Dropcasting	R.T	----	2,24%RH	2-50%	13
PVA/NFC/r-GO	Pour drying	R.T.		0,37%RH	30-98%	14
Cellulose/r-GO	Pour casting	23ºC	40%	--	35-90%	15
PDA/r-GO	Drop casting	25ºC	20%	--	0-97%	16
Graphene/PVP	Ink-jet printing	R.T.	---	0,21-0,3%RH	10-80%	17
r-GO/PVP	Spary coating	20-60ºC	7%	---	7-97.3%	18
r-GO/NTEG	Solution functionalization	20-24ºC	31%	0,33%RH	2-97%	19
Graphene-PHEA	CVD- in situ polimerization	25ºC	29,7%	0,31%RH	0-94%	This work

Here, $S_R(\%)$ is defined as a percentage of the variation in resistance divided by the initial resistance,

$$S_R(\%) = \frac{R_F - R_I}{R_I} \cdot 100$$

Here, $S_R(\%)$ is defined as a percentage of the variation in resistance divided by the initial resistance where R_F is the final resistance and R_I is the initial resistance.

and $S_H(\%)$ is defined as a percentage of the variation in resistance divided by the initial resistance multiplied by the change in relative humidity.

$$S_H(\%) = \frac{R_F - R_I}{R_I \cdot (RH_F - RH_I)} \cdot 100$$

References

1. Ferrari, A. C.; Basko, D. M., Raman spectroscopy as a versatile tool for studying the properties of graphene. *Nature Nanotechnology* **2013,** *8* (4), 235-246.
2. Eckmann, A.; Felten, A.; Mishchenko, A.; Britnell, L.; Krupke, R.; Novoselov, K. S.; Casiraghi, C., Probing the Nature of Defects in Graphene by Raman Spectroscopy. *Nano Letters* **2012,** *12* (8), 3925-3930.
3. Elias, D. C.; Nair, R. R.; Mohiuddin, T. M. G.; Morozov, S. V.; Blake, P.; Halsall, M. P.; Ferrari, A. C.; Boukhvalov, D. W.; Katsnelson, M. I.; Geim, A. K.; Novoselov, K. S., Control of Graphene's Properties by Reversible Hydrogenation: Evidence for Graphane. *Science* **2009,** *323* (5914), 610-613.
4. Bueno, R.; Marciello, M.; Moreno, M.; Sánchez-Sánchez, C.; Martinez, J. I.; Martinez, L.; Prats-Alfonso, E.; Guimerà-Brunet, A.; Garrido, J. A.; Villa, R.; Mompean, F.; García-Hernandez, M.; Huttel, Y.; Morales, M. d. P.; Briones, C.; López, M. F.; Ellis, G. J.; Vázquez, L.; Martín-Gago, J. A., Versatile Graphene-Based Platform for Robust Nanobiohybrid Interfaces. *ACS Omega* **2019,** *4* (2), 3287-3297.
5. Smith, A. D.; Elgammal, K.; Niklaus, F.; Delin, A.; Fischer, A. C.; Vaziri, S.; Forsberg, F.; Råsander, M.; Hugosson, H.; Bergqvist, L.; Schröder, S.; Kataria, S.; Östling, M.; Lemme, M. C., Resistive graphene humidity sensors with rapid and direct electrical readout. *Nanoscale* **2015,** *7* (45), 19099-19109.
6. Popov, V. I.; Nikolaev, D. V.; Timofeev, V. B.; Smagulova, S. A.; Antonova, I. V., Graphene-based humidity sensors: the origin of alternating resistance change. *Nanotechnology* **2017,** *28* (35), 355501.
7. Fan, X.; Elgammal, K.; Smith, A. D.; Östling, M.; Delin, A.; Lemme, M. C.; Niklaus, F., Humidity and CO2 gas sensing properties of double-layer graphene. *Carbon* **2018,** *127*, 576-587.
8. Zhen, Z.; Li, Z.; Zhao, X.; Zhong, Y.; Zhang, L.; Chen, Q.; Yang, T.; Zhu, H., Formation of Uniform Water Microdroplets on Wrinkled Graphene for Ultrafast Humidity Sensing. *Small* **2018,** *14* (15), 1703848.
9. Phan, D.-T.; Chung, G.-S., Effects of rapid thermal annealing on humidity sensor based on graphene oxide thin films. *Sensors and Actuators B: Chemical* **2015,** *220*, 1050-1055.
10. Zaharie-Butucel, D.; Digianantonio, L.; Leordean, C.; Ressier, L.; Astilean, S.; Farcau, C., Flexible transparent sensors from reduced graphene oxide micro-stripes fabricated by convective self-assembly. *Carbon* **2017,** *113*, 361-370.
11. Choi, S.-J.; Yu, H.; Jang, J.-S.; Kim, M.-H.; Kim, S.-J.; Jeong, H. S.; Kim, I.-D., Nitrogen-Doped Single Graphene Fiber with Platinum Water Dissociation Catalyst for Wearable Humidity Sensor. *Small* **2018,** *14* (13), 1703934.
12. Wang, X.; Xiong, Z.; Liu, Z.; Zhang, T., Exfoliation at the Liquid/Air Interface to Assemble Reduced Graphene Oxide Ultrathin Films for a Flexible Noncontact Sensing Device. *Advanced Materials* **2015,** *27* (8), 1370-1375.
13. Tao, J.; Wang, Y.; Xiao, Y.; Yao, P.; Chen, C.; Zhang, D.; Pang, W.; Yang, H.; Sun, D.; Wang, Z.; Liu, J., One-step exfoliation and functionalization of graphene by hydrophobin for high performance water molecular sensing. *Carbon* **2017,** *116*, 695-702.

14. Xu, S.; Yu, W.; Yao, X.; Zhang, Q.; Fu, Q., Nanocellulose-assisted dispersion of graphene to fabricate poly(vinyl alcohol)/graphene nanocomposite for humidity sensing. *Composites Science and Technology* **2016,** *131*, 67-76.
15. Chen, Y.; Pötschke, P.; Pionteck, J.; Voit, B.; Qi, H., Smart cellulose/graphene composites fabricated by in situ chemical reduction of graphene oxide for multiple sensing applications. *Journal of Materials Chemistry A* **2018,** *6* (17), 7777-7785.
16. He, J.; Xiao, P.; Shi, J.; Liang, Y.; Lu, W.; Chen, Y.; Wang, W.; Théato, P.; Kuo, S.-W.; Chen, T., High Performance Humidity Fluctuation Sensor for Wearable Devices via a Bioinspired Atomic-Precise Tunable Graphene-Polymer Heterogeneous Sensing Junction. *Chemistry of Materials* **2018,** *30* (13), 4343-4354.
17. Santra, S.; Hu, G.; Howe, R. C. T.; De Luca, A.; Ali, S. Z.; Udrea, F.; Gardner, J. W.; Ray, S. K.; Guha, P. K.; Hasan, T., CMOS integration of inkjet-printed graphene for humidity sensing. *Scientific Reports* **2015,** *5* (1), 17374.
18. Su, Y.; Xie, G.; Wang, S.; Tai, H.; Zhang, Q.; Du, H.; Zhang, H.; Du, X.; Jiang, Y., Novel high-performance self-powered humidity detection enabled by triboelectric effect. *Sensors and Actuators B: Chemical* **2017,** *251*, 144-152.
19. Anichini, C.; Aliprandi, A.; Gali, S. M.; Liscio, F.; Morandi, V.; Minoia, A.; Beljonne, D.; Ciesielski, A.; Samorì, P., Ultrafast and Highly Sensitive Chemically Functionalized Graphene Oxide-Based Humidity Sensors: Harnessing Device Performances via the Supramolecular Approach. *ACS Applied Materials & Interfaces* **2020,** *12* (39), 44017-44025.
